# SARS-CoV-2 Reinfections in Health-Care Workers, 1 March 2020–31 January 2023

**DOI:** 10.3390/v15071551

**Published:** 2023-07-14

**Authors:** Luca Cegolon, Greta Magnano, Corrado Negro, Francesca Larese Filon

**Affiliations:** 1 Department of Medical, Surgical & Health Sciences, University of Trieste, 34129 Trieste, Italy; gmagnano@units.it (G.M.); negro@units.it (C.N.); larese@units.it (F.L.F.); 2 Occupational Medicine Unit, University Health Agency Giuliano-Isontina (ASUGI), 34129 Trieste, Italy

**Keywords:** SARS-CoV-2 reinfection, COVID-19, health-care workers, vaccine effectiveness

## Abstract

**Objective:** To study SARS-CoV-2 reinfections in health-care workers (HCWs) of the University Health Agency Giuliano-Isontina (ASUGI), covering the provinces of Trieste and Gorizia (northeastern Italy) routinely screened for SARS-CoV-2 via nasopharyngeal swab. **Design:** Cohort study of HCWs (N = 8205) followed since the start of the pandemic (1 March 2020) through 31 January 2023. The risk of reinfection during the Omicron transmission period (after 30 November 2021) among HCWs previously infected by SARS-CoV-2 was estimated based on days since last dose of COVID-19 vaccine received, adjusting for age, sex, job task, workplace, number of doses of COVID-19 vaccines and number of swab tests performed. In the crude as well as adjusted incidence rate analysis, reinfections occurring 15+ days after a first dose of COVID-19 vaccine or 8+ days following a second or more dose were counted. **Results:** In a highly vaccinated population, during the entire study period (1 March 2020–31 January 2023) 5253 HCWs incurred at least one SARS-CoV-2 infection, 4262 HCWs were infected only once, and 1091 were reinfected. Reinfections almost entirely (99.1% = 1071/1091) occurred after 30 November 2021, peaking in July 2022 (N = 161). Six hundred eighty-three reinfections followed a pre-Omicron primary event against 408 reinfections following an Omicron event. Reinfections during the Omicron transmission period occurred a mean of 400 ± 220 days after primary SARS-CoV-2 infection; 512 ± 205 days following a pre-Omicron primary event, as opposed to 218 ± 74 days after an Omicron primary infection. Thirty-four hospitalizations were observed, all before the Omicron wave, following 18 (0.4%) primary SARS-CoV-2 infections and 16 (1.5%) reinfections. By excluding events occurring <15 days after a first dose or <8 days after a further dose of COVID-19 vaccine, 605 reinfections followed a pre-Omicron primary event (raw incidence = 1.4 × 1000 person-days) against 404 after a primary Omicron infection (raw incidence = 0.3 × 1000 person-days). Apart from nurse aids (slightly enhanced biological risk) and academic HCWs (remarkably lower risk with pre-Omicron primary events), the effect of occupation in terms of job task and workplace was marginal. Furthermore, whilst the risk of reinfection was lower in males and HCWs < 60 years old following a pre-Omicron primary infection, HCWs aged 30–50 were more likely to be infected after an Omicron primary event. Regardless of timeline of primary SARS-CoV-2 event, the risk of reinfection decreased with higher number of doses of COVID-19 vaccines, being lowest after the second booster. In particular, VE was 16% for one dose, 51% for two doses, 76% for the booster and 92% for the second booster with a pre-Omicron primary SARS-CoV-2 event. The latter figures increased to 72%, 59%, 74% and 93%, respectively, with Omicron primary infections. **Conclusions**: SARS-CoV-2 reinfections were frequent during the Omicron transmission period, though featured by mild or no symptoms. Whilst the impact of occupation on biological risk was relatively marginal, COVID-19 vaccination had the strongest protective effect against reinfection, with a 93% VE by second booster following an Omicron primary infection.

## 1. Background

SARS-CoV-2 reinfections, defined as events sustained by a genetically distinct SARS-CoV-2 strain ≥ 90 days after a primary infection [1], were an immediate a matter of concern from the start of the pandemic, since human coronaviruses are known to cause reinfection regardless of pre-existing humoral immunity [1,2,3].

The first case of COVID-19 reinfection was reported in a 25-year-old male from Washoe County (Nevada, USA), infected by SARS-CoV-2 on 18 April 2020 and reinfected by a genetically different variant on 5 June 2020 after two negative tests undertaken in May 2020 during follow-up, with the second infection symptomatically more severe than the primary [4]. Seventeen cases of genetically confirmed COVID-19 reinfections were identified between 1 January 2020 and 12 October 2020, the majority (69%) presenting with similarly severe, 18.8% with worse and 12.5% with milder clinical pattern than the first episode [5]. The variable degree of severity of reinfections with respect to the primary event in the early phase of the pandemic was confirmed via a systematic review [6].

Reinfections were rather rare before the Omicron wave [7,8,9,10]. For instance, a nationwide study conducted in health-care workers (HCWs) of Mexico during the first pandemic year examined the risk of reinfections—defined as events at 28+ days since primary infection—from March 2020 (start of the pandemic) through June 2020. Out of 99,993 HCWs investigated (8,268,237 person-days at risk in total), the overall risk of reinfection was 0.21% (2.5 × 100,000 person-days), increasing in immune-depressed individuals or those with kidney disorders and reducing if primary infection was mild or HCWs aged > 50 years [11].

As the Omicron variant (B.1.1.529) rapidly spread throughout the world from January 2022 onward, evidence of its increased immune evasiveness emerged [12], with pre-existing immunity clearly less effective against infection than against previous viral strains [13,14,15]. COVID-19 immunity—induced by vaccination or previous infection—tends to wane over time, and original virulent variants of concern (VOCs) were progressively displaced by more contagious Omicron strains [13,16]. Although remarkably more frequent, reinfections during the Omicron transmission period were clinically milder [5], with the hospitalization rate reportedly dropping to 0.3% [17]. For instance, in a study on HCWs at a tertiary care hospital in Sao Paulo (Brazil), conducted from 10 March 2020 through 10 March 2022, 5865 (23%) of total swab tests (N = 25,570) performed due to symptoms consistent with COVID-19 read positive for SARS-CoV-2, with a 5% (=284/5865) rate of reinfection [18]. In latter study reinfections occurred after a median time of 429 days, were all mild and mostly (88%) occurred during the Omicron wave, with a rate of 4.3%, as opposed to 0.8% with previous VOCs [18].

SARS-CoV-2 reinfections were reportedly less likely in vaccinated individuals who had recovered from a previous infection [3,7,19]. Individuals with pre-existing humoral immunity induced by previous SARS-CoV-2 infection and immunized with one or more doses of COVID-19 vaccines were at lower risk to be infected by Wuhan, Alpha or Delta VOCs than unvaccinated recovering patients or vaccinated individuals not previously infected by the virus [12,20,21,22]. In the SIREN cohort of 35,768 HCWs from the UK—26,280 uninfected versus 9488 previously infected by SARS-CoV-2—2747 primary infections versus 210 reinfections were counted [23]. Having received two doses of Comirnaty (Pfizer BioNTech), administered with a short or long interval between them was associated with high short-term protection against SARS-CoV-2 infection (symptomatic or asymptomatic), waning considerably after six months. By contrast, infection-acquired immunity reinforced by vaccination remained elevated over one year after primary infection [23].

According to a systematic review with meta-analysis, 32.4% of Omicron variant-positive patients were estimated to be asymptomatic and were more likely to be vaccinated, young (median age < 20 years), with history of travel and/or infected outside health-care settings [24]. Asymptomatic or flu-like infections inevitably complicate the assessment of the true impact of COVID-19 and vaccine efficacy (VE). Unlike the general population, HCWs are known to be exposed to a higher biological risk [25]. Moreover, mandatory vaccination uptake combined with systematic screening for SARS-CoV-2 make HCWs an optimal target to assess the infection risk in relation to COVID-19 vaccination status.

## 2. Aims

In view of the above, we conducted a longitudinal study to estimate the risk of reinfections among HCWs of the University Health Agency Giuliano-Isontina (ASUGI), Northeastern Italy, from the start of the pandemic (10 March 2020) through 31 January 2023. To the best of our knowledge, no data on reinfection and vaccine effectiveness (VE) against Omicron were available till the end of January 2023.

## 3. Methods

### 3.1. Ethical Aspects

This study was approved by the Italian Medicine Agency (AIFA), the Ethics Committee of the Italian National Institute of Infectious Diseases (INMI) “Lazzaro Spallanzani” and the regional ethics committee (CEUR) of Friuli Venezia Giulia Region (FVG) (Reg N.188/2022).

In compliance with Italian legislation on privacy law, informed consent from study participants was waived since patients’ data routinely collected for health care reasons were managed anonymously within the framework of an approve study protocol. The present cohort of HCWs contributed to the ORCHESTRA database, a European Union (EU) project funded by Horizon 2020. This study followed the Strengthening of Observational Studies in Epidemiology (STROBE) reporting guidelines.

### 3.2. Study Population

This study investigated incidence of SARS-CoV-2 reinfections from 1 March 2020 through 31 January 2023 among HCWs of ASUGI (N = 8205), including 4 hospitals and other public health services within the provinces of Trieste and Gorizia (FVG Region).

### 3.3. Data Collection

The cohort included HCWs employed by ASUGI on a permanent contract. Data included demographic characteristics (sex, age, occupation, and health care department), primary SARS-CoV-2 infection, reinfection, and hospitalization.

Since 15 April 2020, HCWs of ASUGI were tested by Polymerase Chain Reaction (Rt-PCR) with nasopharyngeal swabs weekly or monthly, depending on the level of their occupational biological risk [3]. HCWs were also swab-tested in cases of symptoms consistent with COVID-19 or for contact tracing [1,3].

### 3.4. Study Endpoint

The study investigated the incidence of SARS-CoV-2 reinfections from 1 March 2020 to 31 January 2023. Reinfection was defined as an infection in the same individual confirmed via rt-PCR at least 90 days after the primary event [26].

HCWs testing positive, with asymptomatic or mild/moderate COVID-19, had to observe home isolation for at least 5 days and were allowed to return to work only after a negative swab test confirmed via Rt-PCR. In compliance with guidelines from the USA Centers for Disease Control and Prevention (CDC), HCWs were exempted from screening tests against SARS-CoV-2 for three months following COVID-19 diagnosis in order to avoid possible false positive results [1].

### 3.5. Statistical Analysis

Continuous variables (age, number of swab tests performed, and time between primary SARS-CoV-2 infection and reinfection) were all non-normally distributed. Although we have presented both the respective mean ± standard deviation (SD) as well as the median (interquartile range, IQR), only the Wilcoxon test was used in comparison. Categorical variables were expressed as number and percentage of HCWs and compared by chi-square tests.

HCWs were followed up from the time of their last vaccine dose through eventual SARS-CoV-2 reinfection, thereby calculating person-days at risk among individuals previously infected. Follow up time for unvaccinated HCWs started on 1 December 2021 instead. Crude incidence rates of reinfections were calculated by explanatory factor and timeline of primary SARS-CoV-2 infection (before 1 December 2021 versus after 30 November 2021). HCWs re-infected before the first COVID-19 vaccine dose (N = 5), all following a pre-Omicron SARS-CoV-2 event, were included among those unvaccinated.

A multivariable Cox proportional hazard regression model was then fitted to investigate risk factors for SARS-CoV-2 reinfection from 1 December 2021 onward. Moreover, the latter analysis was also broken down by timeline of primary SARS-CoV-2 infection (before 1 December 2021 versus after 30 November 2021). Results were expressed as adjusted hazard ratio (aHR) with 95% confidence interval (95% CI). VE against SARS-CoV-2 reinfections was estimated by number of doses of COVID-19 vaccines received (VE = 1-aHR).

Cases with missing values were excluded and complete case analysis was performed.

Statistical analyses were performed using STATA 16.0 (StataCorp LLC, College Station, TX, USA).

## 4. Results

Table 1 reports the characteristics of the study population (N = 8205) by total number of SARS-CoV-2 infections (only one vs. two). Over the entire study period, 4262 (51.9%) HCWs were infected only once, versus 1,091 (13.3%) reinfected.

As can been seen from Table 1, 818 (10%) HCWs were never immunized with a COVID-19 vaccine, 79 (1.0%) received only one dose, 530 (6.5%) two doses, 5988 (73.0%) three doses, 773 (9.4%) four, and 17 (0.2%) five. Moreover, reinfections were less frequent in HCWs older than 60, yet more prevalent in HCWs who were employed in community services and/or unvaccinated (17.2% re-infected unvaccinated vs 5.2% infected once and unvaccinated). The vast majority of HCWs were vaccinated with Comirnaty (Pfizer BioNTech) and with three doses (73.0%). The mean number of swab tests performed during the omicron transmission period was higher in HCWs re-infected (16.2 ± 8.0 vs. 14.4 ± 8.5, respectively; *p* < 0.001; median 15 (IQR = 11; 20) versus 13 (IQR = 8; 19). Thirty-four hospitalizations were observed, all before the Omicron wave, following 18 (0.4%) cases of primary SARS-CoV-2 infections versus 16 (1.5%) re-infections (*p* < 0.001) (Table 1).

**Figure 1:**
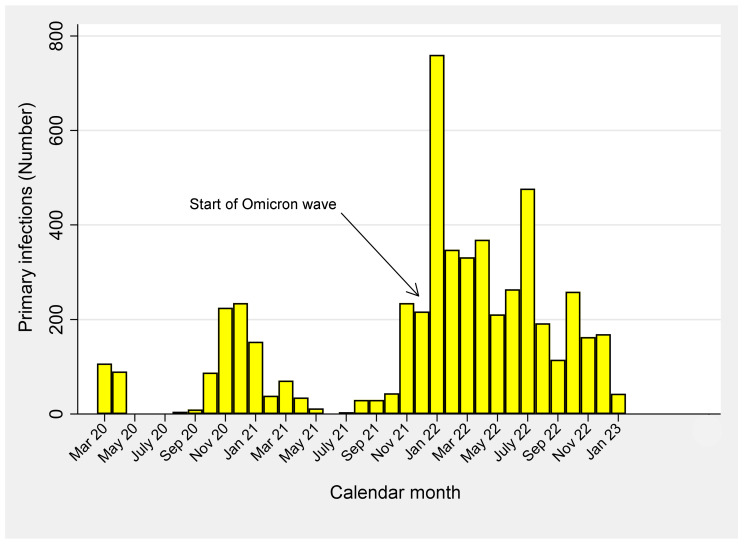
Frequency distribution of primary SARS-CoV-2 infections over time, 1 March 2020–31 January 2023.

Figure 1 reports the frequency distribution of primary SARS-CoV-2 infections over time. As can be seen, the surge of primary infections in autumn 2020 (October through December 20) was followed by a sharp decline in January 2021 and a drop from February 2021 onward, in coincidence with the start of the COVID-19 vaccination campaign. Subsequently, in a highly vaccinated population such as HCWS of ASUGI, primary SARS-CoV-2 infections dramatically re-surged in November 2021, peaking in January 2022, declining thereafter, but maintaining levels of infections considerably higher than pre-Omicron waves and peaking again in July 2022.

**Figure 2:**
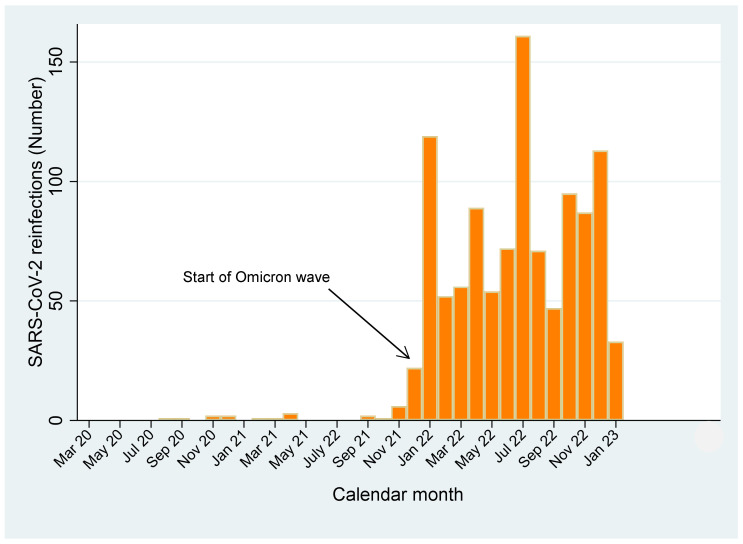
Frequency distribution of SARS-CoV-2 reinfections over time, 1 March 2020–31 January 2023.

Figure 2 shows the frequency distribution of reinfections over time. As can be seen, reinfections almost entirely (99.1% = 1071/1091) occurred after 30 November 2021, peaking in July 2022 (N = 161), in coincidence with maximum surge of primary infections. Six hundred sixty-three reinfections followed a pre-Omicron primary event versus 408 reinfections after an Omicron event.

**Figure 3:**
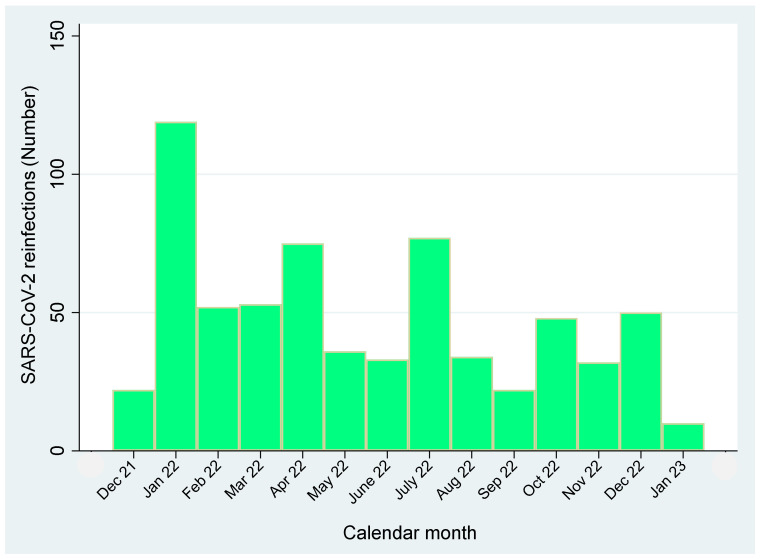
Frequency distribution of SARS-CoV-2 reinfections over time, 90+ days following a pre-Omicron primary event (i.e., before 1 December 2021).

Figure 3 shows the frequency distribution of SARS-CoV-2 reinfections from 1 December onward, following a pre-Omicron primary event (before 1 December 2021). It can be noted that these reinfections were mainly responsible for the first peak in January 2022, progressively declining thereafter.

**Figure 4:**
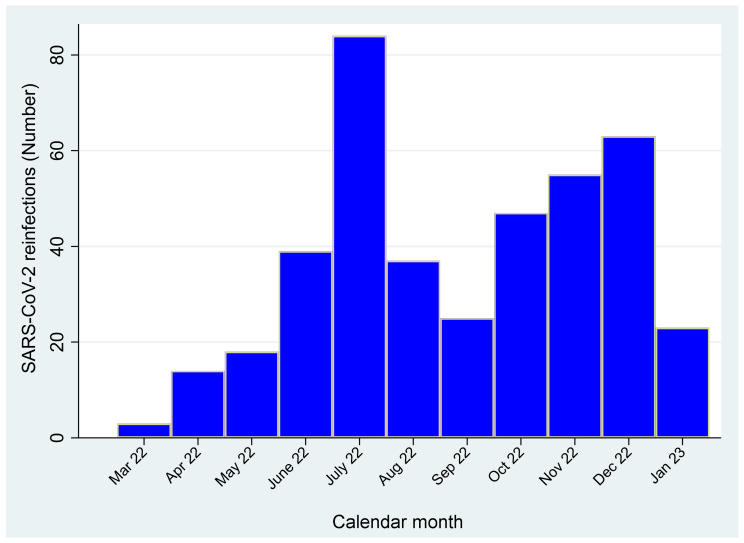
Frequency distribution of SARS-CoV-2 reinfections over time, 90+ days following an Omicron primary event (i.e., after 30 November 2021).

Figure 4 shows the frequency distribution of reinfections from 1 December onward, following an Omicron primary event (after 30 November 2021). As can be seen, the frequency of these reinfections exhibited an opposite trend compared to those following a pre-Omicron primary event, progressively increasing over time, much contributing to the second peak of reinfections observed in July 2022.

**Figure 5:**
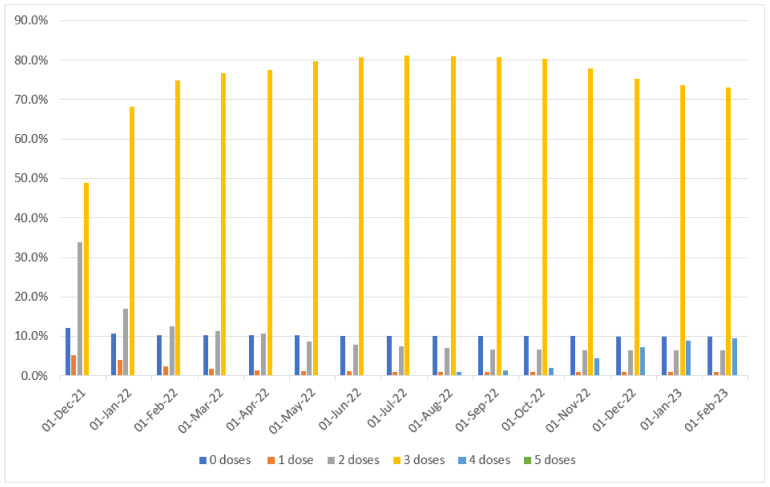
COVID-19 vaccine uptake of health care workers of ASUGI (N = 8205) over time (1 December 2021–31 January 2023), by number of doses received.

Table 2 and Figure 5 report COVID-19 vaccine uptake over time by calendar month since 1 December 2021 through 31 January 2023. Only the 4th and 5th dose were a bivalent vaccine. Whilst the first booster (3rd dose) was still the Wuhan-Hu-1 vaccine, the second and third booster (4th and 5th dose respectively) were bivalent COVID-19 vaccines. Ninety-one-point-two-percent (91.2%) of HCWs were immunized with Comirnaty (Pfizer BioNTech) at third dose. Under Italian law, unvaccinated HCWs were suspended from work or re-reassigned to job tasks not involving patient contact until the end of December 2022. As can be seen from Table 2, at the beginning of the Omicron transmission period, 48.9% (4015/8205) of HCWs were immunized with the booster, 33.8% (2773/8205) with two doses, 5.2% (=428/8205) with one dose, and 12.1% (989/8205) were unvaccinated. By the end of the study (31 January 2023), 9.4% (=773/8205) were immunized with the second booster, 73.0% (=5988/8205) just with the first booster, 6.5% (=530/8205) only with two doses, 1.0% (=79/8205) just with one dose, and 10.0% (=818/8205) were unvaccinated.

Table 3 reports days from primary SARS-CoV-2 infection until reinfection by sex and COVID-19 vaccination status. The group of unvaccinated subjects (N = 193) included 188 HCWs never immunized with COVID-19 vaccines and 5 individuals re-infected before the first dose. By excluding events occurring <15 days before the first dose or <8 days before a second or more dose of COVID-19 vaccines, 605 reinfections followed a pre-Omicron primary event. As can be seen, reinfections occurred after a median of 356 (IQR: 202; 567) days after primary infection in the entire cohort of HCWs, earlier in females (median = 425 days; IQR: 272; 643) than males (median = 328; IQR: 193; 536) days. Moreover, reinfections followed a median of 508 (IQR: 379; 664) days after a pre-Omicron primary event against 200 (IQR: 161; 282.5) days after an Omicron primary event, with no difference by sex. Finally, SARS-CoV-2 reinfections occurred considerably earlier among unvaccinated subjects (median = 185 days; IQR: 154; 300) since primary SARS-CoV-2 event) compared to HCWs immunized by one (median = 484.5 days; IQR: 406; 602), two (median = 361; 95% CI: 175; 574.5), three (median = 416; 95% CI: 258; 623.5) or four (median = 575; 95% CI: 306.5; 767) doses of COVID-19 vaccines.

Table 4 shows the crude incidence rates of reinfections by explanatory factors and timeline of primary SARS-CoV-2 infection (before 1 December 2021 vs. after 30 November 2021). The total number of reinfections decreased from 1091 to 1009, since events occurring <15 days since the first vaccine dose or <8 days since the second or higher dose were not considered in the analysis.

As can be seen, the overall incidence rate was 0.5 (95%CI: 0.5; 0.5) × 1000 P-d, increasing up to 1.4 (95%CI: 1.3; 1.5) × 1000 P-d if the primary event occurred prior to 1 December 2021 and diminishing to 0.3 (95%CI: 0.2; 0.3) × 1000 P-d if the primary event occurred during the Omicron transmission period (after 30 November 2021). The latter pattern was consistently observed for each explanatory factor. Among unvaccinated HCWs, the incidence rate of reinfection was 1.5 (95%CI: 1.3; 1.7) × 1000 P-d, increasing to 2.2 (95%CI: 2.1; 3.3) × 1000 P-d for primary events preceding the Omicron transmission period and declining to 1.0 (95%CI: 0.8; 1.2) × 1000 P-d afterwards. Compared to unvaccinated HCWs, the crude incidence rate of reinfection was consistently lower after any dose of vaccine received, regardless of the occurrence of the primary infection, and it was lowest for 4 doses of COVID-19 vaccine, in the entire cohort [crude incidence rate= 0.1 (95%CI: 0.1; 0.2) × 1000 P-d] or whether primary SARS-CoV-2 infection occurred before [crude incidence rate= 0.3 (95%CI: 0.2; 0.6) × 1000 P-d] or during the Omicron transmission period [crude incidence rate = 0.1 (95%CI 0.0; 0.1) x 1,000 P-d].

Table 5 displays a multivariable Cox regression model for the risk of SARS-CoV-2 reinfection from 1 December 2021 through 31 January 2023 by timeline of primary infection (before vs. during Omicron transmission period). As can be noted, the risk of reinfection was significantly lower in males (aHR = 0.84; 95% CI: 0.73; 0.98), especially in cases of Omicron primary infection (aHR = 0.57; 95% CI: 0.43; 0.75) and lower in HCWs aged 60+ years (aHR = 0.53; 95% CI: 0.38; 0.74), confirmed if primary SARS-CoV-2 infection preceded 1 December 2021 (aHR = 0.55; 95% CI: 0.36; 0.85), though not afterwards. By contrast, if the primary SARS-CoV-2 infection was after 30 November 2021, the risk of reinfection increased in HCWs aged 30–39 years (aHR = 1.63; 95% CI: 1.10; 2.41) or 40–49 years (aHR = 1.51; 95C: 1.02; 2.24). In terms of job task, the risk of reinfection was significantly and remarkably lower for academic HCWs regardless of the timeline of primary infection (aHR = 0.20; 95% CI: 0.03; 0.63) or in case primary events occurring before 1 December 2021 (aHR = 0.08; 95% CI: 0.01; 0.61). Furthermore, in the entire cohort, the risk of reinfection was slightly higher for nurse aids (aHR = 1.38; 95% CI: 1.07; 1.77), but the latter result was not confirmed in the analysis by timeline of occurrence of primary SARS-CoV-2 infection (before or after 1 December 2021). The risk of reinfection was not associated with workplace, but significantly and strongly decreased with number of doses of COVID-19 vaccine, with a clear trend (*p* < 0.001), with stronger effect size in case of omicron primary infection.

In the entire cohort, compared with unvaccinated HCWs, aHR of reinfection was 0.84 (95% CI: 0.56; 1.25) in those immunized with 1 dose, 0.49 (95% CI: 0.38; 0.63) in those immunized with 2 doses, 0.24 (95% CI: 0.20; 0.28) with 3, and 0.08 (95% CI: 0.05; 0.14) with 4 dose, for a VE of 16%, 41%, 76%, and 92%, respectively.

In cases with pre-Omicron primary event (hence before 1 December 2021), the aHR of reinfection was equal to 0.60 (95% CI: 0.38; 0.94) for HCWs who had received 1 vaccine dose, 0.31 (95% CI: 0.22; 0.42) for 2 doses, 0.38 (95% CI: 0.30; 0.48) for 3 doses, and 0.11 (95% CI: 0.05; 0.22) for 4 doses, for a VE of 40%, 69%, 62%, and 89%, respectively.

In case of Omicron primary events (hence after 30 November 2021), the aHR of reinfection was 0.28 (95% CI; 0.09; 0.88) for 1 dose, 0.41 (95% CI: 0.27; 0.63) for 2 doses, 0.16 (95% CI: 0.13; 0.20) for 3 doses and 0.07 (95% CI: 0.04; 0.15) for 4 doses, for a VE of 72%, 59%, 84%, and 93%, respectively.

**Figure 6:**
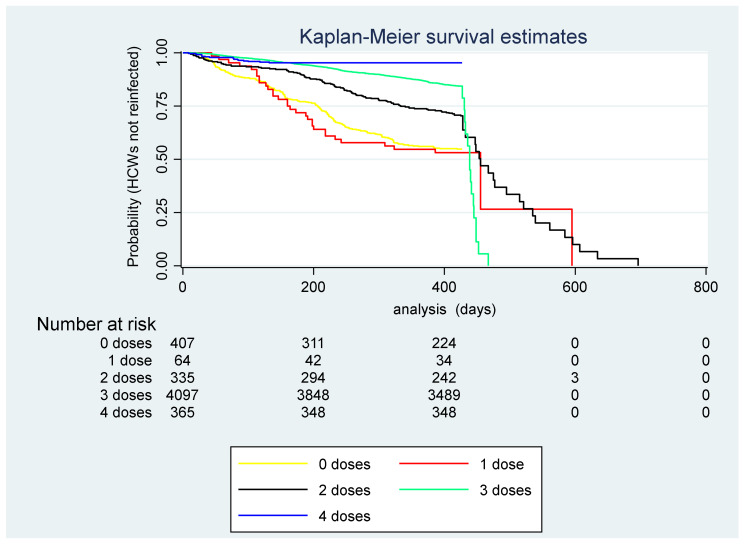
(Kaplan Meier survival curve). Unadjusted risk of SARS-CoV-2 reinfections (N = 1091) during the entire study period (1 March 2020–31 January 2023) by number of doses of COVID-19 vaccine received. Number of health care workers (HCWs) at risk by number of doses of COVID-19 vaccine.

Figure 6 shows the Kaplan-Meier curve for the unadjusted risk of SARS-CoV-2 reinfections in HCWs of ASUGI, by number of doses of COVID-19 vaccine received.

## 5. Discussion

### 5.1. Main Findings

By 1 December 2021, 12.1% of HCWs of ASUGI were unvaccinated, 5.2% were immunized with one dose, 33.8% with two doses and 48.9% with three doses. By the end of the study (31 January 2023), the rate of unvaccinated HCWs was 10%, 1.0% were immunized with one dose, 6.5% with two doses, 73.0% with three doses, 9.4% with four doses, and 0.2% with five.

During the entire study period (1 March 2020–31 January 2023), 5253 HCWs incurred at least one SARS-CoV-2 infection, 4262 HCWs were infected only once, and 1091 were reinfected. Almost the entirety (99.1%) of reinfections occurred after 30 November 2021, peaking in July 2022 (N = 161). Out of total 8,205 HCWs, 683 reinfections followed a pre-Omicron primary event, versus 408 reinfections after an Omicron event.

Reinfections during the Omicron transmission period occurred after a mean of 400 ± 220 days after primary SARS-CoV-2 infection, 512 ± 205 days following a pre-Omicron primary event, versus 218 ± 74 days after an Omicron primary infection. Thirty-four hospitalizations were observed, all occurring before the Omicron wave, following 18 (0.4%) primary infections, versus 16 (1.5%) admissions after a re-infection (*p* < 0.001).

The raw incidence of re-infections was 0.5 × 1000 P-d in the entire cohort, increasing to 1.4 × 1000 P-d if primary SARS-CoV-2 infection occurred before the Omicron transmission period, diminishing to 0.3 × 1000 P-d afterwards.

Apart from nurse aids (slightly enhanced biological risk) and academic HCWs (remarkably lower risk after a pre-Omicron primary event), the effect of occupation in terms of job task and workplace was marginal. Furthermore, whilst the risk of reinfection was lower in males and HCWs <60 years old following a pre-Omicron primary infection, HCWs aged 30–50 years were more likely to be infected after an Omicron primary event.

Regardless of timeline of primary SARS-CoV-2 event, the risk of reinfection decreased with higher number of doses of COVID-19 vaccines, being lowest for the second booster. In the entire cohort, VE was 16%, 51%, 76%, and 92% for one, two, three, and four doses of COVID-19 vaccine, respectively.

Moreover, in case of pre-Omicron primary SARS-CoV-2 event VE was 40% for one dose, 69% for two doses, 62% for the first booster, and 89% for the second booster. The latter figures increased to 72%, 59%, 84%, and 93%, respectively after an Omicron primary infection.

### 5.2. Interpretation of Findings

The higher communicability combined with a lower virulence of Omicron compared with previous VOC has been well established due to its ability to evade neutralizing antibody responses [16,27,28]. Likewise, SARS-CoV-2 reinfections were associated only with mild–moderate disease in the present study.

The raw incidence of reinfections was found to be much higher following a pre-Omicron primary event (1.4 × 1000) in the present study, diminishing afterwards (0.3 × 1000).

COVID-19 vaccination status was the main protective factor against reinfection during the Omicron transmission period in the present study, with a risk progressively decreasing with higher number of doses of COVID-19 vaccine, especially if primary SARS-CoV-2 infection occurred after 30 November 2021.

With pre-Omicron primary events, VE against SARS-CoV-2 reinfection increased from 40% for one dose up to 62% for the first booster and 89% with the second booster. If the primary SARS-CoV-2 event occurred after 30 November 2021, VE increased to 72% for one dose, 84% for the first booster and 93% for the second booster.

In a population base study conducted in Mexico from 3 March 2020 until 13 August 2022, 231,202 cases of SARS-CoV-2 reinfections were recorded, mostly occurring during the Omicron transmission period (89.8% = 207,623/231,202) and in unvaccinated individuals (41.6%). The respective case fatality rate was 0.22%, and vaccination protected against reinfection in the latter study, regardless of the temporal order of the immunizing event [29]. In another study from Mexico City conducted between 1 March 2020 and 28 February 2022, only 73 (5.6%) SARS-CoV-2 reinfections were noted out of 1388 total COVID-19 cases, including 97.3% individuals immunized with 2 doses of COVID-19 vaccine [30]. The overall rate of reinfection in the latter study was 0.231 per 1000 during the Omicron wave, a figure very close to that of the present study (0.3 × 1000). By contrast, higher rates of SARS-CoV-2 reinfections were noted among 11,474 HCWs from New Delhi (India) during the Omicron wave, with an incidence of 4.56 (95% CI: 4.29; 4.85) × 1000 p-d [31].

A systematic review and meta-analysis of 65 studies from 19 countries published before 31 Sep 2022 estimated the risk of SARS-CoV-2 reinfection against primary infection [32]. The high level of protection against reinfections observed with more virulent pre-Omicron VOC (Wuhan, Alpha, and Delta) decreased significantly under Omicron, although protection against severe COVID-19 remained consistently high. Despite waning over time, protection conferred by primary SARS-CoV-2 event against reinfection was at least as high as or even higher than a complete COVID-19 vaccination cycle (2 doses) of mRNA vaccines [32].

Protection against reinfection in previously SARS-CoV-2-infected individuals decreases with time since last immunizing event, regardless of whether any vaccine dose was received before or after infection [19]. However, protection from hybrid immunity acquired by vaccination combined with natural infection was reportedly higher than infection alone or vaccine-only [12,21,33,34,35,36], especially with Omicron but also for previous VOC [19]. In a nationwide study conducted in Israel in August and September 2021, when Delta VOC was predominant, protection against reinfection by previous SARS-CoV-2 infection was higher than that provided by a second dose of vaccination among previously uninfected individuals following the same timeline since last immunizing event. However, a single dose of COVID-19 vaccine after infection reinforced protection against secondary infection [19].

A study was conducted on HCWs in Quebec (Canada) between 27 March and 4 June 2022 to examine the risk of reinfection in relation to vaccination status. Of 37,732 presumed BA.2 cases recorded, 2,521 (6.7%) were reinfections after pre-Omicron primary SARS-CoV-2 events, versus 659 (1.7%) reinfections following primary infection occurring during Omicron [36]. Pre-Omicron primary SARS-CoV-2 events were associated with a 38% reduction of BA.2 infection, further increasing to 56%, 69%, and 70% among HCWs immunized with one, two, or three doses of COVID-19 vaccines respectively. Higher protection (72%) against BA.2 infection was found with primary SARS-CoV-2 infection during the Omicron transmission period, further increasing to 96% in HCWs also immunized with 2 doses of COVID-19 vaccines. However, the booster dose did not further enhance protection (96%), questioning the additional benefit of further boosters doses against future SARS-CoV-2 variants in case of hybrid humoral immunity [36].

Differently from previous investigations in the same cohort [3,15], the biological risk was only marginally influenced by occupation task in the present study. The risk of reinfection was in fact slightly higher only for nurse aids in the entire cohort and remarkably lower for academic clinical tasks in case of primary SARS-CoV-2 infection occurred before Omicron. Likewise, the biological risk was not influenced by health care premises in the present study, suggesting extra-occupational spread of asymptomatic or mild–moderate SARS-CoV-2 infections in a highly vaccinated population of HCWs during the Omicron transmission period. The progressive lifting of mandatory health protection measures in the community allowed the spread of the SARS-CoV-2 outside health care premises, where face masks and social distancing were not systematically enforced any longer.

Lower risk of reinfection in males in the present study may be explained by their lower prevalence in patient-facing tasks; in fact, 78% of 2908 nurses and 79.3% of 1233 nurse aids were females. However, this estimate is in contrast with a previous investigations in the same cohort, reporting higher risk of SARS-CoV-2 infection and severe disease in males [3,15]. Likewise, lower risk of reinfection for HCWs older than 60 years, particular after pre-Omicron primary events, may be explained by the lower proportion of HCWs > 60 years assigned to patient-facing tasks [3,15].

### 5.3. Strengths and Weaknesses

This study investigated a large cohort (N = 8205) of highly vaccinated HCWs subject to systematic screening for SARS-CoV-2, from the start of the pandemic until 31 January 2023. This approach allowed the detection of asymptomatic HCWs and those developing symptoms after swab testing, allowing subsequent isolation to contain the spread of COVID-19 infection in health care premises.

However, our study has some limitations. First of all, although this cohort was systematically screened and followed up throughout the pandemic, without serology information it is impossible to exclude the possibility that some HCWs categorized as singly infected may have had undetected prior COVID-19 infection instead.

Moreover, we did not have information on individuals’ risk factors for COVID-19, especially co-morbidities, which can influence the incidence rate of SARS-CoV-2 infection. Another limitation is the lack of data on COVID-19 symptoms, since only hospitalization was recorded.

Finally, this was a dynamic cohort, with a few HCWs who may have retired and some other may have been employed during the study period. However, this should have had only a limited impact on the calculation of the number of unvaccinated HCWs (by subtraction from the total number of HCWs, N=8205), since date of COVID-19 vaccination and date of SARS-CoV-2 infection were still available for retirees and newly employed HCWs from the Regional Data Registry of FVG, regardless of whether the HCW was in service during the study period.

## 6. Conclusions

In a highly vaccinated population of 8205 HCWs, 1091 cases of reinfection were notified since the start of the pandemic (1 March 2020) through 31 January 2023, almost entirely (99.1%) occurring after 30 November 2021 and peaking in July 2022. Six-hundred eighty-three reinfections occurred following a pre-Omicron primary event (raw incidence = 1.4 × 1000 P-d), versus 408 reinfections after an Omicron primary infection (raw incidence = 0.3 × 1000 P-d).

The risk of reinfection was only marginally influenced by occupation.

Regardless of timeline of primary SARS-CoV-2 event, the risk of reinfection decreased with higher number of doses of COVID-19 vaccines, being lowest for the second booster. In particular, in the entire cohort VE was 16%, 51%, 76%, and 92% for one, two, three and four doses of COVID1-9 vaccine, respectively. In case of pre-Omicron primary SARS-CoV-2 infection, VE was 40% for one dose, 69% for two doses, 62% for the first booster, and 89% for the second booster. The latter figures increased to 72%, 59%, 84%, and 93%, respectively, in case of reinfections following an Omicron primary SARS-CoV-2 events.

Humoral immunity following natural SARS-CoV-2 infection should be taken into account by COVID-19 vaccination policies for HCWs and the general population.

## Figures and Tables

**Table 1:** SARS-CoV-2 infections (singleton vs double) of health care workers of ASUGI, by explanatory factors. Number (N), column percentage (%) and *p*-value of chi-square (for categorical terms) or Wilcoxon test (for continuous terms). A&E = Accident and Emergency.

Terms				Total	SARS-CoV-2 Infections		*p*-Value	
					Only One	Two		
**TOTAL (row %)**				8205 (100)	4262 (51.9)	1091 (13.3)		
**Primary SARS-CoV-2** **Infections**		**Before 1 December 2021**		1434 (26.8)	751 (17.6)	683 (62.6)	<0.001	
		**After 30 November 2021**		3919 (73.2)	3511 (82.4)	408 (37.4)		
**SARS-CoV-2** **Reinfections**		**Before 1 December 2021**		20 (1.8)	NA	20		
		**After 30 November 2021**		1071 (98.2)	NA	1071		
**Sex**	**Females**			5572 (67.9)	2954 (60.3)	809 (74.1)	<0.001	
	**Males**			2633 (32.1)	1308 (30.7)	282 (25.9)		
**Age** (years)	**Mean** **± SD**			46.6 ± 12.0	46.3 ± 11.7	44.9 ± 10.9		
	**Median (IQR)**			48 (36; 57)	48 (36; 56)	46 (36; 54)	<0.001	
	**<30**			866 (10.6)	451 (10.6)	115 (10.5)	<0.001	
	**30–39**			1738 (21.2)	889 (21.2)	258 (23.7)		
	**40–49**			1725 (21.0)	952 (21.0)	290 (26.6)		
	**50–59**			2851 (34.8)	1365 (34.8)	367 (33.6)		
	**60+**			1025 (12.5)	605 (12.5)	61 (5.6)		
**Employment**	**Administrative clerks**			1401 (17.1)	615 (14.4)	131 (12.0)	<0.001	
	**Medical doctors**			1203 (14.7)	659 (15.5)	107 (9.8)		
	**Nurses**			2908 (35.4)	1604 (37.6)	465 (42.6)		
	**Academics**			263 (3.2)	76 (1.8)	6 (0.6)		
	**Nurse aids**			1233 (15.0)	658 (15.4)	233 (21.4)		
	**Technicians**			324 (4.0)	165 (3.9)	42 (3.9)		
	**Health technicians**			873 (10.6)	485 (11.4)	107 (9.8)		
**Dpt.**	**Administrative services**			1195 (14.4)	584 (13.7)	116 (10.1)	<0.001	
	**Medical and geriatric ward**			2617 (31.9)	1237 (29.0)	323 (12.3)		
	**Surgical ward**			1199 (14.6)	692 (16.2)	142 (13.0)		
	**Community services**			1636 (19.9)	890 (20.9)	251 (23.0)		
	**A&E**			764 (9.3)	426 (10.0)	149 (13.7))		
	**Health services (radiology, labs)**			593 (7.2)	322 (7.6)	73 (6.7))		
	**Non-clinical work and other**			115 (1.4)	56 (1.3)	18 (15.7)		
	**COVID-19 ward**			86 (1.1)	55 (1.3)	19 (1.7))		
**N. doses of COVID-19 vaccine**	**0**			818 (10.0)	223 (5.2)	188 (17.2)	<0.001	
	**1**			79 (1.0)	32 (0.8)	33 (3.0)	<0.001	
	**2**			530 (6.5)	215 (5.0)	135 (12.4)	<0.001	
	**3**			5988 (73.0)	3441 (80.7)	688 (63.1)	<0.001	
	**4**			773 (9.4)	348 (8.2)	47 (4.3)	<0.001	
	**5**			17 0.2)	3 (0.1)	0	-	
**Swabs tests** **(Number)**	**1 March 2020–31 January 2023** **(range: 5–130)**		**Mean** **± SD**	37.8 ± 19.9	41.2 ± 18.4	47.4 ± 18.0		
			**Median (IQR)**	37 (23: 51)	40 (28; 53)	46 (35; 58)		<0.001
	**1 December 2021–31 January 2023** **(range: 2–70)**		**Mean** **± SD**	12.9 ± 8.8	14.4 ± 8.5	16.2 ± 8.0		
			**Median (IQR)**	12 (6–18)	13 (8–19)	15 (11; 20)		<0.001
**1st Vaccine Dose**	**Comirnaty**			6875 (94.6)	3785 (95.1)	832 (92.5)		0.005
	**Spikevax**			319 (4.4)	161 (4.1)	58 (6.5)		
	Others *			71 (1.0)	34 (0.8)	7 (1.0)		
**2nd Vaccine Dose**	**Comirnaty**			6594 (94.9)	3664 (95.9)	686 (92.0)		<0.001
	**Spikevax**			298 (4.3)	139 (3.6)	54 (7.2)		
	Others *			56 (0.8)	16 (0.5)	5 (0.8)		
**3rd Vaccine Dose**	**Comirnaty**			6095 (91.2)	3473 (91.7)	658 (89.5)		0.116
	**Spikevax**			558 (8.4)	274 (7.2)	64 (8.7)		
	Others *			28 (0.4)	42 (1.1)	13 (1.8)		

* Nuvaxoid, Jannsen, Vaxzevria.

**Table 2:** Number of health care workers of ASUGI (Total=8205) by number of doses of COVID-19 vaccine received over time (1 December 2021- 31 January 2023).

DATE	0 Doses	1 Dose	2 Doses	3 Doses	4 Doses	5 Doses
**1 December 2021**	989	428	2773	4015	0	0
**15 December 2021**	920	362	1695	5228	0	0
**1 January 2022**	887	323	1394	5601	0	0
**15 January 2022**	873	259	1240	5833	0	0
**1 February 2022**	853	195	1022	6135	0	0
**15 February 2022**	847	158	944	6256	0	0
**1 March 2022**	847	139	922	6297	0	0
**15 March 2022**	849	127	903	6326	0	0
**1 April 2022**	845	116	877	6361	6	0
**15 April 2022**	842	108	806	6443	6	0
**1 May 2022**	840	99	708	6548	10	0
**15 May 2022**	837	95	668	6594	11	0
**1 June 2022**	833	90	644	6625	13	0
**15 June 2022**	832	89	630	6638	16	0
**1 July 2022**	831	84	617	6656	17	0
**15 July 2022**	830	82	594	6671	28	0
**1 August 2022**	830	79	581	6641	74	0
**15 August 2022**	987	77	537	6503	101	0
**1 September 2022**	829	77	553	6626	120	0
**15 September 2022**	829	77	548	6623	128	0
**1 October 2022**	828	77	543	6597	160	0
**15 October 2022**	817	77	537	6503	260	0
**1 November 2022**	824	78	533	6398	371	1
**15 November 2022**	822	78	532	6296	473	4
**1 December 2022**	818	79	532	6178	589	9
**15 December 2022**	818	79	531	6111	655	11
**1 January 2023**	818	79	531	6037	728	12
**15 January 2023**	818	79	531	6007	756	14
**31 January 2023**	818	79	530	5988	773	17

**Table 3:** Time (days) from primary SARS-CoV-2 infection until reinfection occurred during the omicron transmission period (1 December 2021–31 January 2023) in health care workers (HCWs) of ASUGI, by timeline of primary SARS-CoV-2 event (pre-Omicron vs. Omicron), sex (males vs. females) and last dose of COVID-19 vaccine received. Unvaccinated included also HCWs re-infected before the first COVID-19 vaccine dose (N = 5). Mean (M) ± standard deviation (SD); median, interquartile range (IQR); Wilcoxon test *p*-value.

TERM			MALES	FEMALES	TOTAL	*p*-Value
**All HCWs** (N = 1071)		**Range**	100; 1010	92; 1025	92; 1025	
		**M ± SD**	450.7 ± 227.6	382.7 ± 215.3	400.3 ± 220.5	
		**Median (IQR)**	425 (272; 643)	328 (193; 536)	356 (202; 567)	<0.001
**Primary** **SARS-CoV-2** **event**	**Pre-Omicron** (N = 683)	**Range**	100; 1010	92; 1025	92; 1025	
		**M ± SD**	535.8 ± 206.8	502.1 ± 204.3	512.3 ± 205.5	
		**Median (IQR)**	524 (396; 687)	497.5 (371; 655)	508 (379; 664)	0.069
	**Omicron** (N = 408)	**Range**	104; 378.3	92; 373	92; 378.3	
		**M ± SD**	225.7 ± 76.8	216.6 ± 73.3	218.3 ± 74.0	
		**Median (IQR)**	205 (172; 287.5)	199.5 (159; 281.7)	200 (161; 282.5)	0.388
**Vaccination** **status**	**Unvaccinated** (N = 193)	**Range**	100; 744	92; 857	92; 857	
		**M ± SD**	249.4 ± 149.9	282.7 ± 167	239.6 ± 143.1	
		**Median (IQR)**	185 (154; 300)	242 (151; 390)	181 (154; 294)	0.143
	**>14 days after 1st dose and** **before 2nd dose** (N = 158)	**Range**	300; 753.54	101; 981	101; 981	
		**M ± SD**	530.3 ± 125.3	499.9 ± 147.9	505.8 ± 141.7	
		**Median (IQR)**	503 (413; 620)	479 (405; 598.5)	484.5 (406; 602)	0.397
	**>7 days after 2nd dose and** **Before 3rd dose** (N = 136)	**Range**	104; 802	97; 800	(97; 802	
		**M ± SD**	424 ± 217.7	363.4 ± 206.3	379.1 ± 210.2	
		**Median (IQR)**	425 (248; 633)	360 (171; 541)	361 (175; 574.5)	0.161
	**>7 days after 3rd dose** **and before 4th dose** (N = 680)	**Range**	108.3; 1010	92; 1025	92; 1025	
		**M ± SD**	496.7 ± 225.1	424.5 ± 219.6	443.7 ± 223.2	
		**Median (IQR)**	484 (315; 676)	379 (246; 590)	416 (258; 623.5)	<0.001
	**>7 days after 4th dose** **and before 5th dose** (N = 16)	**Range**	263; 935	276; 1000	263; 1000	
		**M ± SD**	625.2 ± 329.7	541.2 ± 246.3	567.5 ± 266.5	
		**Median (IQR)**	704 (279; 932)	539 (318; 752)	575 (306.5; 767)	0.827

**Table 4:** Crude incidence rates of SARS-CoV-2 reinfections during 1 December 2021–31 January 2023 in health care workers (HCWs) of ASUGI previously infected by SARS-CoV-2 at least once (N= 5253), by explanatory factors and time period of primary SARS-CoV-2 infection (before 1 December 2021 vs. after 30 November 2021). Number of cases (N), person-days at risk (P-d) since the last vaccine dose and raw incidence (cases × 1000 P-d) with 95% confidence interval (95% CI). Dpt. = Department; A&E = Accident and Emergency.

TERMS			SARS-CoV-2 Reinfections								
			All (1 December 2021–31 January 2023)			Primary SARS-CoV-2 Infection before 1 December 2021			Primary SARS-CoV-2 Infection after 30 November 2021		
			Cases (N)	P-d	Cases × 1000 P-d (95% CI)	Cases (N)	P-d	Cases × 1000 P-d (95% CI)	Cases (N)	P-d	Cases × 1000 P-d (95% CI)
**All HCWs**			1009	2,048,703.1	0.5 (0.5; 0.5)	605	434,168.5	1.4 (1.3; 1.5)	404	1,614,534.6	0.3 (0.2; 0.3)
**Sex**	**Females**		755	1,434,299.5	0.5 (0.5; 0.6)	425	292,239.5	1.5 (1.3; 1.6)	330	1,142,060	0.3 (0.3; 0.3)
	**Males**		254	614,403.1	0.4 (0.4; 0.5)	180	141,929.1	1.3 (1.1; 1.5)	74	472,474.5	0.2 (0.1; 0.2)
**Age** (years)	**<30**		105	214,568.3	0.5 (0.4; 0.6)	71	47,003	1.5 (1.2; 1.9)	34	167,565.3	0.2 (0.1; 0.3)
	**30–39**		236	435,343.2	0.5 (0.5; 0.6)	131	99,979	1.3 (1.1; 1.6)	105	335,464.2	0.3 (0.3; 0.4)
	**40–49**		271	467,634.7	0.6 (0.5; 0.7)	159	103,209.5	1.5 (1.3; 1.8)	112	364,425.2	0.3 (0.3; 0.4)
	**50–59**		341	720,223	0.5 (0.4; 0.5)	210	139,682	1.5 (1.3; 1.7)	131	580,541	0.2 (0.2; 0.3)
	**60+**		56	210,833.9	0.3 (0.2; 0.3)	34	44,295	0.8 (0.5; 1.1)	22	166,538.9	0.1 (0.1; 0.2)
**Job** **task**	**Administrative clerks**		122	291272.5	0.4 (0.4; 0.5)	68	48,691.5	1.4 (1.1; 1.8)	54	242,581	0.2 (0.2; 0.3)
	**Medical doctors**		97	303,653.8	0.3 (0.3; 0.4)	66	59,747	1.1 (0.9; 1.4)	31	243906.8	0.1 (0.1; 0.2)
	**Nurses**		432	782,791.7	0.6 (0.5; 0.6)	255	176,082.1	1.4 (1.3; 1.6)	177	606,764.6	0.3 (0.3; 0.3)
	**Nurse aids**		3	33,035	0.1 (0.0; 0.3)	1	5586	0.2 (0.0; 1.3)	2	27,449	0.1 (0.0; 0.3)
	**Academics**		211	327,992.7	0.6 (0.6; 0.7)	139	87,369	1.6 (1.3; 1.9)	72	240,450.7	0.3 (0.2; 0.4)
	**Health technicians**		40	78,958.5	0.5 (0.4; 0.7)	24	17,072	1.4 (0.9; 2.1)	16	61,886.5	0.3 (0.2; 0.4)
	**Technicians**		104	230,944	0.5 (0.4; 0.5)	52	39,448	1.3 (1.0; 1.7)	52	191,496	0.3 (0.2; 0.4)
**Dpt**.	**Administrative services**		110	273,524	0.4 (0.3; 0.5)	63	45,848	1.4 (1.1; 1.8)	47	227,676	0.2 (0.2; 0.3)
	**Medical and geriatric ward**		294	594,444.7	0.5 (0.4; 0.6)	177	143,007	1.2 (1.1; 1.4)	117	451,437.7	0.1 (0.1; 0.2)
	**Surgical ward**		132	324,351	0.4 (0.3; 0.5)	83	61,235	1.4 (1.1; 1.7)	49	263,116	0.1 (0.1; 0.2)
	**Community services**		235	434,933.1	0.5 (0.5; 0.6)	128	88,390.5	1.4 (1.2; 1.7)	107	346,542.6	0.2 (0.2; 0.2)
	**A&E**		132	210,566	0.6 (0.5; 0.7)	90	50,856	1.8 (1.4; 2.2)	42	159,710.0	0.2 (0.1; 0.2)
	**Health services (radiology, labs)**		71	154,940.9	0.5 (0.4; 0.6)	42	28,510	1.5 (1.1; 2.0)	29	126,429.9	0.1 (0.1; 0.2)
	**Non-clinical unit and other**		16	27,678	0.6 (0.4; 0.9)	8	6197	1.3 (0.6; 2.6)	8	21,481	0.2 (0.1; 0.4)
	**COVID-19 unit**		19	28,265.3	0.7 (0.4; 1.1)	14	10,124	1.4 (0.8; 2.3)	5	18,141.3	0.2 (0.1; 0.5)
**Doses of** **COVID-19 vaccine**		**0**	193	129,391.5	1.5 (1.3; 1.7)	98	36,134	2.2 (2.1; 3.3)	95	93,257.5	1.0 (0.8; 1.2)
		**1**	29	19,606	1.5 (1.0; 2.1)	25	14,692	1.7 (1.1; 2.5)	4	4914	0.8 (0.3; 2.2)
		**2**	118	123,654	1.0 (0.8; 1.1)	76	62,066	1.2 (1.0; 1.5)	42	61,588	0.7 (0.5; 0.9)
		**3**	652	1,625,118.6	0.4 (0.4; 0.4)	397	293,277.5	1.4 (1.2; 1.5)	255	1,333,841.1	0.2 (0.2; 0.2)
		**4**	17	149,652	0.1 (0.1; 0.2)	9	27,999	0.3 (0.2; 0.6)	8	121,653	0.1 (0.0; 0.1)

**Table 5:** Cox proportional regression model for the risk of SARS-CoV-2 reinfections regardless of timeline of primary SARS-CoV-2 infection or by time period of primary event (before 1 December 2021 vs. after 30 November 2021). Adjusted hazard ratio (aHR) with 95% confidence interval (95% CI). Obs = complete analysis observations. Dpt. = Department; A&E = Accident and Emergency. Heat-map: green highlights mark significantly lower risks of reinfection; orange highlights mark significantly higher risks of reinfection.

Terms			Multivariable Cox Regression Analysis aHR (95% CI)		
			All Reinfections (5271 obs.)	With Primary Event before 1 December 2021 (1356 obs.)	With Primary Event after 30 November 2021 (3915 obs.)
**Sex**		**Females**	Reference	Reference	Reference
		**Males**	0.84 (0.73; 0.98)	0.95 (0.79; 1.15)	0.57 (0.43; 0.75)
**Age** (years)		**<30**	Reference	Reference	Reference
		**30–39**	1.11 (0.88; 1.41)	0.84 (0.62; 1.13)	1.63 (1.10; 2.41)
		**40–49**	1.13 (0.90; 1.43)	0.91 (0.68; 1.22)	1.51 (1.02; 2.24)
		**50–59**	0.85 (0.67; 1.06)	0.89 (0.67; 1.18)	0.94 (0.64; 1.39)
		**≥** **60**	0.53 (0.38; 0.74)	0.55 (0.36; 0.85)	0.58 (0.33; 1.02)
**Employment**	**Administrative clerks**		Reference	Reference	Reference
	**Medical doctors**		0.90 (0.67; 1.21)	0.91 (0.61; 1.35)	0.70 (0.43; 1.11)
	**Nurses**		1.22 (0.96; 1.53)	0.88 (0.63; 1.23)	1.24 (0.87; 1.76)
	**Academics**		0.20 (0.06; 0.63)	0.08 (0.01; 0.61)	0.32 (0.08; 1.33)
	**Nurse Aids**		1.38 (1.07; 1.77)	0.94 (0.65; 1.34)	1.23 (0.83; 1.80)
	**Technicians**		1.27 (0.87; 1.86)	1.02 (0.61; 1.71)	1.25 (0.66; 2.35)
	**Health technicians**		0.96 (0.72; 1.28)	0.83 (0.54; 1.28)	1.09 (0.72; 1.65)
**Dpt.**	**Administrative services**		Reference	Reference	Reference
	**Community services**		1.09 (0.84; 1.39)	0.83 (0.54; 1.28)	1.18 (0.81; 1.72)
	**Health services (radiology, labs)**		0.87 (0.66; 1.17)	0.81 (0.57; 1.16)	0.90 (0.58; 1.42)
	**Surgical wards**		1.10 (0.85; 1.41)	0.79 (0.53; 1.18)	1.33 (0.91; 1.95)
	**Medical and geriatric wards**		1.29 (0.97; 1.71)	1.07 (0.72; 1.59)	1.19 (0.75; 1.90)
	**COVID-19 wards**		1.12 (0.81; 1.55)	1.07 (0.67; 1.71)	1.07 (0.65; 1.76)
	**A&E**		1.17 (0.68; 2.01)	0.87 (0.39; 1.92)	1.50 (0.69; 3.27)
	**Non-clinical units**		1.33 (0.80; 2.22)	0.78 (0.42; 1.47)	0.86 (0.32; 2.27)
**Doses of COVID-19 vaccine**	**0**		Reference	Reference	Reference
	**1**		0.84 (0.56; 1.25)	0.60 (0.38; 0.94)	0.28 (0.09; 0.88)
	**2**		0.49 (0.38; 0.63)	0.31 (0.22; 0.42)	0.41 (0.27; 0.63)
	**3**		0.24 (0.20; 0.28)	0.38 (0.30; 0.48)	0.16 (0.13; 0.20)
	**4**		0.08 (0.05; 0.14)	0.11 (0.05; 0.22)	0.07 (0.04; 0.15)
**Swab tests during Omicron wave** (linear term)			1.00 (1.00; 1.03)	1.04 (1.03; 1.05)	1.00 (1.00; 1.03)

## Data Availability

The data generated and analyzed during the current study are not publicly available, since they were purposively collected by the authors for the present study, but they are available from the corresponding author on reasonable request.
